# Supplementary material for: Lacking Immunotherapy Biomarkers for Biliary Tract Cancer: A Comprehensive Systematic Literature Review and Meta-Analysis
Source: Cells. 2023 Aug 19;12(16):2098. doi: 10.3390/cells12162098 (PMC10453268; doi:10.3390/cells12162098)
Supplement: Supplementary file 1 [file cells-12-02098-s001.zip › cells-2506026-supplementary.pdf]

Suppl. Tables

Suppl Table S1. Summary of studies on PD-L1 expression in BTCs.

PMID	Title	First Author	Journal	Study reliability for our purpose	Study design	Study tipology	No. Pts	iCCA	pCCA	dCC	GBC	AVC	Cut-off	Antibody	Number of positive for PDL1	Number of patients evaluable for PDL1	Percentage of positive
35022908	A real-world study of the efficacy and safety of anti-PD-1 antibodies plus lenvatinib in patients with advanced gallbladder cancer	Zuo B et al. 2022 [41]	Cancer Immunol Immunother	Poor	Retrospective	Monocentric	31	n/a	n/a	n/a	31	n/a	n/a	n/a	13	31	41.9
33884649	Molecular Characterization of Biliary Tract Cancer Predicts Chemotherapy and Programmed Death 1/Programmed Death-Ligand 1 Blockade Responses	Yoon JG et al. 2021 [42]	Hepatology	Poor	Retrospective	Monocentric	121	33	35		41	12	TPS ≥1%	22C3	22*	48	45.8

34526987	CTLA-4 Synergizes With PD1/PD-L1 in the Inhibitory Tumor Microenvironment of Intrahepatic Cholangiocarcinoma	Guo XJ et sl. 2021 [43]	Front Immunol	Medium	Retrospective	Monocentric	290	290	n/a	n/a	n/a	n/a	PD-L1 >5% positive staining	n/a	93*	290	32.1*
34253205	Relationship between PD-L1 expression, CD8+ T-cell infiltration and prognosis in intrahepatic cholangiocarcinoma patients	Deng M et al. 2021 [44]	Cancer Cell Int	Medium	Retrospective	Monocentric	69	69	n/a	n/a	n/a	n/a	≥ 2% of cancer cells	Proteintech	8	69	11.6
33912461	The Efficacy and Safety of Apatinib Plus Camrelizumab in Patients With Previously Treated Advanced Biliary Tract Cancer: A Prospective Clinical Study	Wang D et al. 2021 [45]	Front Oncol	Good	Prospective	Monocentric	21	15	4		2	n/a	≥ 1% of tumor cells and ≥ 1% of tumor-associated immune cells	22C3	4	21	19.0
33918309	Programmed Death Ligand-1 (PD-L1) Is an Independent	Albrecht T et. al 2021 [46]	Cancers (Basel)	Medium	Retrospective	Monocentric	129	n/a	n/a	n/a	131	n/a	CPS>1	SP263	45*	129	34.9

	Negative Prognosticator in Western-World Gallbladder Cancer															
33212090	Clinicopathologic features, tumor immune microenvironment and genomic landscape of Epstein-Barr virus-associated intrahepatic cholangiocarcinoma	Huang YH et al. 2021 [47]	J Hepatol	Medium	Retrospective	Monocentric	303	303	n/a	n/a	n/a	n/a	immunoreactivity scoring system (IRS)	n/a	82	303	27.1
33077921	Prognostic impact of tumor microvessels in intrahepatic cholangiocarcinoma: association with tumor-infiltrating lymphocytes	Yugawa K et al. 2021 [48]	Mod Pathol	Medium	Retrospective	Monocentric	100	100	n/a	n/a	n/a	n/a	≥ 1% of total cancer cells	E1L3N	49	100	49.0
33712681	Obesity is a risk factor for intrahepatic cholangiocarcinoma progression associated with	Yugawa K et al. 2021 [49]	Sci Rep	Medium	Retrospective	Monocentric	74	74	n/a	n/a	n/a	n/a	≥ 1% of total cancer cells	E1L3N	41	74	55.4

	alterations of metabolic activity and immune status																
33442265	Clinicopathological and Prognostic Significance of Immunoscore and PD-L1 in Intrahepatic Cholangiocarcinoma	Wu H et al. 2021 [50]	Onco Targets Ther	Medium	Retrospective	Monocentric	50	50	n/a	n/a	n/a	n/a	≥5% of cancer cells	Abcam	8	50	16.0
33730723	Programmed Death Ligand 1 Expression as a Prognostic Marker in Patients with Advanced Biliary Tract Cancer	Kim H et al. 2021 [51]	Oncology	Medium	Retrospective	Monocentric	186	72	90		24	n/a	CPS≥1	22C3	53	186	28.5
33328484	Genomic profiling reveals high frequency of DNA repair genetic aberrations in gallbladder cancer	Abdel-Wahab R et al. 2020 [52]	Sci Rep	Good	Retrospective	Multicentric	760	n/a	n/a	n/a	n/a	n/a	>1% tumor cell	SP142	13	83	15.7
33228682	PD-1/PD-L1 expression profiles within intrahepatic cholangiocarcinoma	Tian L et al. 2020 [53]	World J Surg Oncol	Medium	Retrospective	Monocentric	322	322	n/a	n/a	n/a	n/a	estimated Youden index of PD-1/PD-L1 expression	n/a	80	322	24.8

	predict clinical outcome																
33239315	Machine learning: an approach to preoperatively predict PD-1/PD-L1 expression and outcome in intrahepatic cholangiocarcinoma using MRI biomarkers	Zhang J et al. 2020 [54]	ESMO Open	Good	Retrospective	Monocentric	98	98	n/a	n/a	n/a	n/a	n/a	n/a	32	98	32.7
33014052	Somatic Mutation Profiling of Intrahepatic Cholangiocarcinoma: Comparison between Primary and Metastasis Tumor Tissues	Xu SF et al. 2020 [55]	J Oncol	Medium	Retrospective	Multicentric	202	202	n/a	n/a	n/a	n/a	TPS>3%	n/a	5	44	11.4
32173382	Molecular classification and therapeutic targets in extrahepatic cholangiocarcinoma	Montal R et al. 2020 [56]	J Hepatol	Good	Retrospective	Multicentric	189	n/a	189		n/a	n/a	membranous staining of tumor cells or stromal cells in >1%	Abcam	52	162	32.1

32040618	Prognostic impact of CD8+ T cell distribution and its association with the HLA class I expression in intrahepatic cholangiocarcinoma	Asahi Y et al. 2020 [57]	Surg Today	Medium	Retrospective	Monocentric	69	69	n/a	n/a	n/a	n/a	≥ 5% of the tumor cells' membranes	E1L3N	10	69	14.5
32631434	Comprehensive molecular profiling of intrahepatic cholangiocarcinoma in the Chinese population and therapeutic experience	Wang L et al. 2020 [58]	J Transl Med	Medium	Retrospective	Monocentric	122	122	n/a	n/a	n/a	n/a	percentage of membrane staining on TCs or ICs in the overall area of the tumor, regardless of intensity	SP263	20*	122	16.4
32028754	Programmed death-ligand 1 expression and its correlation with clinicopathological parameters in gallbladder cancer	Kim JH et al. 2020 [59]	J Pathol Transl Med	Medium	Retrospective	Monocentric	101	n/a	n/a	n/a	101	n/a	>1% tumor cell	SP263	19	101	18.8
31410753	Expression of Programmed Death Ligand 1 Is	Dong Z et al. 2020 [60]	Dig Dis Sci	Medium	Retrospective	Monocentric	125	125	n/a	n/a	n/a	n/a	n/a	Cell Signaling	52	125	41.6

	Associated with the Prognosis of Intrahepatic Cholangiocarcinoma																
31407173	Clinical Significance of PD-L1 Expression in Both Cancer and Stroma Cells of Cholangiocarcinoma Patients	Kitano Y et al. 2020 [61]	Ann Surg Oncol	Medium	Retrospective	Monocentric	177	114	63		n/a	n/a	≥25% of the cancer cells	E1L3N	54	177	30.5
31949927	Patterns and genomic correlates of PD-L1 expression in patients with biliary tract cancers	Mody K et al. 2019 [62]	J Gastrointest Oncol	Good	Retrospective	Multicentric	652	372	77		203	n/a	≥2+ and/or ≥5% on tumor cells	SP142	56	652	8.6
31081949	Programmed cell death ligand-1 (PD-L1) expression in extrahepatic biliary tract cancers: a comparative study using 22C3, SP263 and E1L3N anti-PD-L1 antibodies	Ahn S et al. 2019 [12]	Histopathology	Medium	Retrospective	Monocentric	183	n/a	89	94	n/a	n/a	>1% tumor cell	22C3 (SP263) (E1L3N)	31	183	16.9

31392046	Molecular profiling of biliary cancers reveals distinct molecular alterations and potential therapeutic targets	Weinberg BA et al. 2019 [63]	J Gastrointest Oncol	Good	Retrospective	Multicentric	1502	356	116		238	n/a	Staining was scored for intensity and percentage	SP142	63	798	7.9
31109808	Nivolumab alone or in combination with cisplatin plus gemcitabine in Japanese patients with unresectable or recurrent biliary tract cancer: a non-randomised, multicentre, open-label, phase 1 study	Ueno M et al. 2019 [64]	Lancet Gastroenterol Hepatol	Good	Prospective	Multicentric	30	11	7		10	2	≥1% of tumour cells	pharmDx 28–8	13	57	22.8
31367249	Distinct PD-L1/PD1 Profiles and Clinical Implications in Intrahepatic Cholangiocarcinoma Patients with Different Risk Factors	Lu JC et al. 2019 [65]	Theranostics	Medium	Retrospective	Monocentric	320	320	n/a	n/a	n/a	n/a	>5% positive area in tumor cells	SP142	99	320	30.9

30885276	HHLA2 in intrahepatic cholangiocarcinoma: an immune checkpoint with prognostic significance and wider expression compared with PD-L1	Jing CY et al. 2019 [66]	J Immunother Cancer	Medium	Retrospective	Monocentric	153	153	n/a	n/a	n/a	n/a	≥5% tumor cells or ≥1% immune cells	E1L3N	43	153	28.1
30773525	Programmed death ligand-1, tumor infiltrating lymphocytes and HLA expression in Chinese extrahepatic cholangiocarcinoma patients: Possible immunotherapy implications	Yu F et al. 2019 [67]	Biosci Trends	Medium	Retrospective	Monocentric	62	n/a	17	45	n/a	n/a	TIS score, with a score ≥ 3 being defined as PD-L1 positivit	E1L3N	20	62	32.3
30646854	Programmed cell death ligand 1 (PD-L1, CD274) in cholangiocarcinoma - correlation with clinicopathological	Kriegsmann M et al. 2019 [68]	BMC Cancer	Medium	Retrospective	Monocentric	170	72	57	41	n/a	n/a	>1% tumor cell	SP263	19	170	11.2

	data and comparison of antibodies															
30377340	Mismatch repair deficiency is a rare but putative therapeutically relevant finding in non-liver fluke associated cholangiocarcinoma	Goeppert B et al. 2019 [69]	Br J Cancer	Medium	Retrospective	Monocentric	308	159	106	43	n/a	n/a	n/a	n/a	19	144	13.2
30323667	Programmed death ligand 1 expression in human intrahepatic cholangiocarcinoma and its association with prognosis and CD8(+) T-cell immune responses	Zhu Y et al. 2018 [70]	Cancer Manag Res	Medium	Retrospective	Monocentric	192	192	n/a	n/a	n/a	n/a	≥5% membranous PD-L1 expression on tumor cells	n/a	34	192	17.7
29882997	Clinical relevance of PD-L1 expression in gallbladder cancer: a potential target for therapy	Neyaz A et al. 2018 [71]	Histopathology	Medium	Retrospective	Monocentric	174	n/a	n/a	n/a	174	n/a	TPS ≥1%	SP263	40	174	23.0
30055582	Classification of gallbladder cancer by	Lin J et al. 2018 [72]	BMC Cancer	Medium	Retrospective	Monocentric	66	n/a	n/a	n/a	66	n/a	≥5% in tumor cells	E1L3N	12	66	18.2

	assessment of CD8(+) TIL and PD-L1 expression																
29805739	Prognostic value of CD8CD45RO tumor infiltrating lymphocytes in patients with extrahepatic cholangiocarcinoma	Kim R et al. 2018 [73]	Oncotarget	Medium	Retrospective	Monocentric	34	n/a	44		n/a	n/a	cutoff value of 5%	5H1	10	34	29.4*
29732001	Prognostic impact of programmed cell death ligand 1 (PD-L1) expression and its association with epithelial-mesenchymal transition in extrahepatic cholangiocarcinoma	Ueno T et al 2018 [74]	Oncotarget	Medium	Retrospective	Monocentric	117	n/a	70	47	n/a	n/a	H-score and cut-offs: 1%, 5%, 10% or 50 of stained tumor	SP142 and E1L3N	10	117	8.5
28419539	PD-L1 expression in extrahepatic cholangiocarcinoma	Walter D et al. 2017 [75]	Histopathology	Poor	Retrospective	Monocentric	69	n/a	40	29	n/a	n/a	semiquantitative score based on percentage and intensity	E1L3N	8	69	11.6
28693161	PD-L1 and PD-1 expression correlate	Ma K et al. 2017 [13]	Oncol Lett	Medium	Retrospective	Monocentric	70	n/a	70		n/a	n/a	0, <5% stained cells; 1, 5-25%	ab174838	30	70	42.9

	with prognosis in extrahepatic cholangiocarcinoma											stained cells; 2, 26-50% stained cells; and 3, >50% stained cells				
28670887	Programmed Death-Ligand 1 (PD-L1) Expression Associated with a High Neutrophil/Lymphocyte Ratio in Cholangiocarcinoma	Sangkhamanon S et al. 2017 [76]	Asian Pac J Cancer Prev	Poor	Retrospective	Monocentric	46	32	13	n/a	n/a	>1% tumor cell membrane	5H1	32	46	69.6
28139862	The PD-1/PD-L1 axis may be aberrantly activated in occupational cholangiocarcinoma	Sato Y et al. 2017 [77]	Pathol Int	Poor	Retrospective	Monocentric	68	23	45	n/a	n/a	PD-L1 in tumor cell categories: negative, <5% positive, 5–10% positive, >10% positive.	28-8	5	68	7.4

Suppl. Table S2. Summary of studies on TMB in BTCs.

PMID	Title	First Author	Journal	Study reliability	Study design	Study typology	No. Pts	ICC	pCC	dCC	GBC	AVC	Cut-off mut/Mb	Mean or median mut/Mb	No. TMB-H	No. Pts evaluable	Percentage TMB-H

				for our purpose													
33884649	Molecular Characterization of Biliary Tract Cancer Predicts Chemotherapy and Programmed Death 1/Programmed Death-Ligand 1 Blockade Responses	Yoon JG et al. 2021 [42]	Hepatology	Poor	Retrospective	Monocentric	121	33	35		41	12	20*	11.9*	9*	121*	7.4*
33387086	Comprehensive analysis of genomic alterations of Chinese hilar cholangiocarcinoma patients	Feng F et al. 2021 [78]	Int J Clin Oncol	Medium	Retrospective	Monocentric	63	n/a	63	n/a	n/a	n/a	20*	3.8	1*	60	1.7
33754015	Mutational spectrum and precision oncology for biliary tract carcinoma	Lin J et al. 2021 [79]	Theranostics	Good	Retrospective	Multicentric	803	475	164		164	n/a	9.36	1.2	33	803	4.1
33535978	Comprehensive analysis of genomic mutation signature and tumor mutation burden for prognosis of intrahepatic cholangiocarcinoma	Zhang R et al. 2021 [80]	BMC Cancer	Good	Retrospective	Multicentric	318	318	n/a	n/a	n/a	n/a	10	1.3	12	318	3.8
33328484	Genomic profiling reveals high frequency of DNA repair genetic aberrations in gallbladder cancer	Abdel-Wahab R et al. 2020[52]	Sci Rep	Good	Retrospective	Multicentric	760	n/a	n/a	n/a	n/a	n/a	19.5	2.6	9	760	1.2
32898339	Integrative clinical and molecular analysis of advanced biliary tract cancers on immune checkpoint blockade reveals potential markers of response	Li J et al. 2020 [81]	Clin Transl Med	Medium	Retrospective	Monocentric	26	11	n/a	n/a	15	n/a	20*	5.6*	1*	17 *	5.9

32631434	Comprehensive molecular profiling of intrahepatic cholangiocarcinoma in the Chinese population and therapeutic experience	Wang L et al. 2020 [58]	J Transl Med	Medium	Retrospective	Monocentric	122	122	n/a	n/a	n/a	n/a	20*	n/a	2*	122*	1.6
32923885	Intrahepatic Cholangiocarcinoma: Genomic Heterogeneity Between Eastern and Western Patients	Cao J et al. 2020 [82]	JCO Precis Oncol	Good	Retrospective	Multicentric	447	447	n/a	n/a	n/a	n/a	10	n/a	26*	321	8.1*
32576609	Molecular profile of BRCA-mutated biliary tract cancers	Spizzo G et al. 2020 [83]	ESMO Open	Good	Retrospective	Multicentric	1292	746	189		353	n/a	17	6.1*	34	1038	3.3*
31949927	Patterns and genomic correlates of PD-L1 expression in patients with biliary tract cancers	Mody K et al. 2019 [62]	J Gastrointest Oncol	Good	Retrospective	Multicentric	652	372	77		203	n/a	17	7.2	19	612	3.1*
31700903	Precision oncology for gallbladder cancer: insights from genetic alterations and clinical practice	Lin J et al. 2019 [84]	Ann Transl Med	Good	Prospective	Monocentric	60	n/a	n/a	n/a	60	n/a	12.5	5.4	9	60	15.0
31392046	Molecular profiling of biliary cancers reveals distinct molecular alterations and potential therapeutic targets	Weinberg BA et al. 2019 [63]	J Gastrointest Oncol	Good	Retrospective	Multicentric	1502	356	116		238	n/a	17	n/a	14	352	4.0
31068370	Alterations in DNA Damage Repair Genes in Primary Liver Cancer	Lin J et al. 2019 [85]	Clin Cancer Res	Medium	Prospective	Monocentric	357	122	n/a	n/a	n/a	n/a	20	4.0$	2	122	1.6

Suppl. Table S3. Summary of studies on MSI/dMMR in BTCs.

PMID	Title	First Author	Journal	Study reliability for our purpose	Study design	Study typology	No. Pts	ICC	pCC	dCC	GBC	AVC	Methods	No. Pts. MSI/dMMR	No. Pts evaluable	MSI/dMMR Percentage
34820003	Low prevalence of biliary tract cancer with defective mismatch repair genes in a Japanese hospital-based population	Ando Y et al. 2022 [86]	Oncol Lett	Medium	Retrospective	Monocentric	116	14	17	32	30	23	IHC/validation PCR	5	116	4.3
34653023	Prognosis of Advanced Cholangiocarcinoma in the Palliative Care Setting: A Series of 201 Cases	Zeng T et al. 2021 [87]	Altern Ther Health Med	Medium	Retrospective	Monocentric	59	110	20	17	54	n/a	PCR	5	59	8.5
33884649	Molecular Characterization of Biliary Tract Cancer Predicts Chemotherapy and Programmed Death 1/Programmed Death-Ligand 1 Blockade Responses	Yoon JG et al. 2021 [42]	Hepatology	Poor	Retrospective	Monocentric	121	33	35		41	12	Targeted sequencing-IHC	1	121	0.8
33998775	Prediction of mismatch repair deficient biliary tract cancer: Role of morphological features and host immune	Suda R et al. 2021 [88]	J Hepatobiliary Pancreat Sci	Medium	Retrospective	Monocentric	662	104	n/a	n/a	n/a	n/a	IHC/validation PCR	15	662	2.3

	response detected by routine hematoxylin-eosin staining															
34367955	Expression of HER2 and Mismatch Repair Proteins in Surgically Resected Gallbladder Adenocarcinoma	Sung YN et al. 2021 [89]	Front Oncol	Poor	Retrospective	Monocentric	216	n/a	n/a	n/a	216	n/a	IHC/validation PCR	3	216	1.4
34124390	Success rate of microsatellite instability examination and complete response with pembrolizumab in biliary tract cancer	Kai Y et al. 2021 [24]	JGH Open	Poor	Retrospective	Monocentric	60	24	12	4	16	n/a	MSI Kit (PCR-based method)	2	60	3.3
33683644	Molecular Profiling and Targeted Therapy in Cholangiocarcinoma: An Observational, Retrospective Multicenter Study	Garcia-Pardo M et al. 2021 [90]	J Gastrointest Cancer	Medium	Retrospective	Multicentric	30	18	8		1	n/a	NGS	1	30	3.3
34113169	Rare DNA Mismatch Repair-Related Protein Loss in Patients with Intrahepatic Cholangiocarcinoma and Combined	Yu J et al. 2021 [91]	Cancer Manag Res	Medium	Retrospective	Monocentric	73	73	n/a	n/a	n/a	n/a	IHC	2	73	2.7

	Hepatocellular-Cholangiocarcinoma and Their Response to Immunotherapy															
33754015	Mutational spectrum and precision oncology for biliary tract carcinoma	Lin J et al. 2021 [79]	Theranostics	Good	Retrospective	Multicentric	803	475	164		164	n/a	whole-exome sequencing (WES) or targeted panel	10	803	1.2
33328484	Genomic profiling reveals high frequency of DNA repair genetic aberrations in gallbladder cancer	Abdel-Wahab R et al. 2020 [52]	Sci Rep	Good	Retrospective	Multicentric	760	n/a	n/a	n/a	760	n/a	NGS	3	551	0.5
33014052	Somatic Mutation Profiling of Intrahepatic Cholangiocarcinoma: Comparison between Primary and Metastasis Tumor Tissues	Xu SF et al. 2020 [55]	J Oncol	Medium	Retrospective	Multicentric	202	202	n/a	n/a	n/a	n/a	NGS	7	184	3.8
32898339	Integrative clinical and molecular analysis of advanced biliary tract cancers on immune checkpoint blockade reveals potential markers of response	Li J et al. 2020 [81]	Clin Transl Med	Medium	Retrospective	Monocentric	26	11	n/a	n/a	15	n/a	NGS	0	17	0.0

32173382	Molecular classification and therapeutic targets in extrahepatic cholangiocarcinoma	Montal R et al. 2020 [56]	J Hepatol	Good	Retrospective	Multicentric	189	n/a	189		n/a	n/a	Whole genome sequencing	6	150	4.0
32631434	Comprehensive molecular profiling of intrahepatic cholangiocarcinoma in the Chinese population and therapeutic experience	Wang L et al. 2020 [58]	J Transl Med	Medium	Retrospective	Monocentric	122	122	n/a	n/a	n/a	n/a	NGS	2	122	1.6
32923885	Intrahepatic Cholangiocarcinoma: Genomic Heterogeneity Between Eastern and Western Patients	Cao J et al. 2020 [82]	JCO Precis Oncol	Good	Retrospective	Multicentric	447	447	n/a	n/a	n/a	n/a	NGS/validation PCR	4	321	1.2
32576609	Molecular profile of BRCA-mutated biliary tract cancers	Spizzo G et al. 2020 [83]	ESMO Open	Good	Retrospective	Multicentric	1292	746	189		353	n/a	IHC/NGS/MSI kit	24	1089	2.4
31844887	Mismatch Repair Protein Deficiency/Microsatellite Instability Is Rare in Cholangiocarcinomas and Associated With Distinctive Morphologies	Ju JY et al. 2020 [23]	Am J Clin Pathol	Medium	Retrospective	Monocentric	96	31	65		n/a	n/a	MSI kit	6	96	6.3
32019287	Efficacy and Safety of Pembrolizumab in Patients with Refractory	Kang J et al. 2020 [29]	Cancer Res Treat	Good	Prospective	Monocentric	40	20	8		12	n/a	IHC	0	40	0.0

	Advanced Biliary Tract Cancer: Tumor Proportion Score as a Potential Biomarker for Response															
32010606	Genomic Features and Clinical Characteristics of Adolescents and Young Adults With Cholangiocarcinoma	Feng H et al. 2020 [92]	Front Oncol	Medium	Retrospective	Monocentric	192	n/a	n/a	n/a	n/a	n/a	NGS	5	192	2.6
31949927	Patterns and genomic correlates of PD-L1 expression in patients with biliary tract cancers	Mody K et al. 2020 [62]	J Gastrointest Oncol	Good	Retrospective	Multicentric	652	372	77		203	n/a	NGS	13	608	2.1
31392046	Molecular profiling of biliary cancers reveals distinct molecular alterations and potential therapeutic targets	Weinberg BA et al. 2019 [63]	J Gastrointest Oncol	Good	Retrospective	Multicentric	1502	356	116		238	n/a	NGS	7	352	2.0
31068370	Alterations in DNA Damage Repair Genes in Primary Liver Cancer	Lin J et al. 2019 [85]	Clin Cancer Res	Medium	Prospective	Monocentric	122	122	n/a	n/a	n/a	n/a	targeted NGS panel	7	122	5.7
31068195	Low frequency of mismatch repair deficiency in gallbladder cancer	Goeppert B et al. 2019 [93]	Diagn Pathol	Medium	Retrospective	Monocentric	69	n/a	n/a	n/a	69	n/a	PCR	1	69	1.4

30377340	Mismatch repair deficiency is a rare but putative therapeutically relevant finding in non-liver fluke associated cholangiocarcinoma	Goeppert B et al. 2019 [69]	Br J Cancer	Medium	Retrospective	Monocentric	308	159	106	43	n/a	n/a	PCR	4	308	1.3
29747443	Microsatellite Instability Occurs Rarely in Patients with Cholangiocarcinoma: A Retrospective Study from a German Tertiary Care Hospital	Winkelmann R et al. 2018 [94]	Int J Mol Sci	Medium	Retrospective	Monocentric	102	35	42	25	n/a	n/a	IHC/PCR	1	102	1.0
28667006	Whole-Genome and Epigenomic Landscapes of Etiologically Distinct Subtypes of Cholangiocarcinoma	Jusakul A et al. 2017 [95]	Cancer Discov	Good	Prospective	Monocentric	71	n/a	n/a	n/a	n/a	n/a	NGS	3	71	4.2
27864690	Infrequent mismatch repair protein loss in gallbladder cancer patients in Japan	Yoshida H et al. 2017 [96]	Virchows Arch	Medium	Retrospective	Monocentric	211	n/a	n/a	n/a	211	n/a	IHC	2	211	0.9
